# Echoes of the Month

**Published:** 1924-11

**Authors:** 


### ECHOES OF THE MONTH.

### ANOTHER CORONER ON INQUEST FEES.

Another coroner, Dr. Waldo, is supporting Dr. Edwin Smith in his criticism of the new regulation of the L.C.C. disallowing fees to doctors of Poor Law institutions for making autopsies. He is of opinion that in the public interest they ought to be paid, and that it is bad policy not to pay them. There might be a tendency for these institution doctors to sign up cases. A doctor might say to himself, "I shall sign up, and there is an end of it. If I don't I shall have to make an unpleasant post-mortem examination and go before the coroner." Dr. Waldo has long contended that the statutory fee of £1 1s. paid to medical men for making a post mortem should be changed to £2 2s. for all doctors alike. This fee is allowed in Australia.

### NATIONAL HEALTH DETERIORATING.

"The health of the nation," said Sir William Milligan, lecturing on "Physique in Relation to National Efficiency," was not improving but deteriorating, and unless far more stringent measures were taken the position of the country in a few years would be terrible. As a direct result of tuberculosis alone it has been estimated that £150,000,000 was lost every year in England and Wales. Fifty per cent. of the existing disease was preventable, and a sure way of increasing the efficiency of the nation was to clear away the slums and to establish more and more laboratories for research. It had been estimated that a healthy male child of ten was worth £200 to the nation and a man between twenty and forty £500, Sir William Milligan stated, adding: "The iniquitous birth control and contraception so popular among all classes in this country will lead to the downfall of the British Empire."

### HOSPITAL AS AN HOTEL.

A delegate arriving late at night during a recent political convention in the United States, finding no available hotel lodging in the city, told the taxi-cab driver to take him to a hospital. He was entered as a patient desiring an operation for appendicitis. As it was late, the usual examinations were postponed until the following morning, when the patient declared that he felt so much better that he thought he would postpone his operation, and called for his bill. Later he boasted to his friends that he had a better bed and breakfast for the modest charge of $3 than his friends had at the various hotels where, at convention rates, the guests were paying $10 a day.

### UNEMPLOYMENT AMONG WOMEN DOCTORS.

It is estimated that there are now 300 women doctors without practices or appointments due to the supply being greater than the demand for their services. Dr. Cox, Secretary of the B.N.A., is of opinion that the position will adjust itself in time. "Nobody yet knows exactly the capacity of the country for absorbing women doctors, and it will take some little time to settle the question. Before long we shall arrive at the right solution. There is no doubt that many of those who qualified at the end of last year are having difficulty in finding employment. What I hope to see is women going into practice and competing on the same terms as men, instead of so many taking up public appointments. We shall never know how far women doctors are a success until we find them in general practice."

### THE SKELETON IN THE CUPBOARD.

Medical students beginning the anatomical part of their education this month are finding, in the words of a scientific store catalogue, that "the supply of bones is still very limited, particularly disarticulated skulls." A good-quality skeleton, "articulated in a superior manner," costs £10 10s. "Best quality ditto, bones well marked, with movable spine," runs to £15 15s.; while the traditional cupboard in which well-conducted families, if not budding doctors, keep their skeleton is an additional £4 15s. A skull such as that which moved Hamlet to his famous soliloquy costs two guineas—that is, third quality; but a first-rate skull, "mounted à Beauchene on a stand, each bone in its correct position with relation to complete skull, under glass shade," is priced at £15 15s. An articulated skull "with good teeth" is £3 3s. A pair of femurs for playful use as cross-bones in decoration costs 15s.; hands or feet articulated on catgut are 10s. 6d. each, while occasionally second-hand half skeletons are obtainable for £2 10s. All these human skeletons and bones come from abroad.

### AN UNUSED DONATION.

In 1878 a grateful patient or friend of the Marine Hospital Service in the United States (now the Public Health Service) gave $523.50 "for the benefit of the Marine Hospital at Bath, Maine." The amount was placed at interest by the Collector of Customs, and is still on deposit, because there was no Marine Hospital at Bath, Maine, and never has been, the nearest ones on that coast being located at Portland, Maine, and Boston, Massachusetts. Practically all cities in the United States are increasing in size, and the city of Bath is no exception to this rule. It is not at all improbable that the time may come when a new Marine Hospital will be needed on the Maine Coast to serve those who go down to the sea in ships, in conformity with the Government policy established in 1798 to provide medical care for merchant seamen.

### PLAGUE CAUSED BY FLEAS.

It is now suggested that the great Plague of London was due to fleas. The bacillus of bubonic plague was found in the blood of fleas by a Japanese scientist in Hongkong a few years ago. Why have there been no more plagues here, while they have been common in India? Since the Plague the brown rat, which lived in houses, has in England been ousted by the black rat, which lives in sewers. Fewer fleas! Moreover, the insanitary housing and street conditions have been swept away. Still fewer fleas!

### FISHERMEN'S STRANGE DISEASE.

The fishermen who follow their calling in the Kurisches Haff, the great landlocked bay in East Prussia, are reported to have recently been suffering from a strange epidemic which has hitherto been quite unknown and experts are unable to diagnose. The disease presents many curious points. In the first place, it develops as a rule in the early morning, when the air is still heavy. It has been noted that men who fish with a line become more frequently infected than net fishermen, and the man at the helm of the boat may escape altogether. Only fishermen and people working in the neighbourhood of the bay catch the disease. The peasants who do not approach the shore remain immune. Also in some places dogs, cats, geese, and ducks have died from it. The epidemic is considered to show some resemblance to poisoning by arsenicated hydrogen and phosphuretted hydrogen. The waste products of the cellulose factories near Königsberg find access to the bay, and the fishermen themselves have observed an interesting phenomenon—that the seaweeds in the bay have flowered for the first time in many years, and that they subsequently decompose, giving off a pungent odour, especially in the early hours of the morning.

### BRITISH SURGICAL INSTRUMENTS.

Speaking recently on surgical instruments, Dr V. Warren-Low said that British manufacturers of surgical instruments had not only kept abreast of the times but had presented to the world types, characteristics, and qualities which not only defied competition but defied imitation. That was true to-day as it had been true in the searching days of the war, when the art of the British surgical instrument maker had reached a standard of excellence the envy of the world.

### BRAIN WORK FOR THE TUBERCULOUS.

Dr. James Crocket, medical superintendent of the Consumption Sanatoria of Scotland at Bridge of Weir, is of opinion that the tuberculous child should not be looked upon as a potential manual labourer. In the majority of instances any form of strenuous work, indoor or out-of-door, would prove to be beyond its powers. Many hundreds of pounds, he stated, had been spent in training tuberculous ex-Service men for occupations like general farmers, poultry farming, pig-keeping, etc., but he had yet to hear of one authentic case of an individual so trained who had been able to make a living at these occupations. Any scheme of education for tuberculous children should be based on the fact that they were not going to make their living by the sweat of their brow. Manual training should, therefore, be given a secondary place in the curriculum, and subjects should be chosen which would develop to the fullest the child's mental faculties so that he might be able to earn his livelihood by brain work. For tuberculous children the school age should be extended to 17 or 18 years, or even longer when circumstances demanded it.

### "DON'TS" FOR HEALTH VISITORS.

Professor Kenwood, of London University, speaking recently to Health Visitors, said that the public official should bear in mind that he or she was an official only and not a tin deity. They should remember that they were the servants of the community. They should be good listeners but not too ready talkers. His "don'ts" for health visitors were: "Don't quizz; don't interfere, and for heaven's sake don't ask one unnecessary question. Don't overwhelm people, and don't lose sight of the human side."

### THE SUFFERINGS OF LOUIS XIV.

An article in the "Manchester Guardian" on "The Torments of Louis XIV." speaks of the sufferings undergone by the Roi Soleil at the hands of his medical men:—"In the year 1695 his doctors pulled out the last tooth remaining in his left upper jaw. The operation, which seems to have lasted for hours, was performed with so little skill that a fragment of bone was accidentally wrenched from the royal palate. There were no anæsthetics in those days, and it is difficult to conceive the torture the King must have suffered. The King was, of course, unable to masticate his food. His doctors ordered a diet of hot spices, peppery stews, and highly-seasoned salads so as to 'stimulate' his injured palate. He developed a voracious appetite. The thirst engendered by pepper and hot spices made him crave for fruit and cooling drinks. He would gorge himself full on figs and melons. On the evening before his death he ate thirty fresh figs and drank a big bowl of iced water."

### THE VOICE AND NERVOUS DISEASES.

The phonographic record is the latest aid to medical science in the diagnosis of nervous diseases. Dr. E. W. Scripture, Professor of Experimental Phonetics in the University of Vienna, lecturing at King's College recently, demonstrated various methods of recording the most minute fluctuations in the human voice, and said these records were playing an increasingly important part in this direction. The only laboratory established for the purpose of making this diagnosis from records is at the West End Hospital for Nervous Diseases.

### NEW INSTRUMENT FOR HEART OPERATIONS.

Drs. Claude S. Beck and Elliott C. Cutler of the Harvard Medical Schools have invented a new instrument to be called a "cardiovalvulotome" for use in operations in cases of valvular disease of the heart in which a valve has become rigid and obstructs the blood stream. When the chest has been opened, the instrument is pushed into the vein leading from the lung to the heart, and by means of a spring in the handle the surgeon is able to cut out the obstructing segment and remove it. Experiments were first made on thirty dogs, of whom twenty-four survived. It was then tried with a human patient with apparently complete success and with little loss of blood.

### WORK AS A TONIC.

It has lately been said that "overwork" is largely an illusion. It is but a pleasant name for unfitness engendered either by too much success or too little resolution. Men with assured positions are notoriously more liable to this complaint than those who must remain in the fighting line or lose all that they possess. The truth is that great success is unhealthy and unwholesome. Man was not created to be prosperous in the permanent and established sense of that word. He was created to strive towards prosperity. Striving and fitness are synonymous terms.

### THE SALE OF BLOOD.

"There is a type of man," says an article on "Blood Transfusion" in the *Birmingham Daily Mail*, "for whom the surrender of a pint or so of blood is, literally, all in the day's work." "I could put my hand, within half an hour, on at least three 'universal donors,'" the writer was told by a surgeon at one of our great hospitals, who has had wide experience in this operation. "Donors (givers of blood) and recipients (the patients) are medically divided into four groups—A, B, C, D. Classes B and C comprise persons who are only suitable to give or receive blood from those of the same class. The A's are 'universal recipients,' who can safely receive blood from any sound donor; and the D's are 'universal donors,' who can furnish blood to any patient, whatever his or her condition. Some of the D's—the universal providers—make quite a good thing out of their physical perfection. Any surgeon who requires a donor has only to approach the Labour Exchange, or the Salvation Army shelter, to find a lusty fellow ready to sell his blood at a reasonable figure. In the old days (the operation was first performed about eighty years ago) 'a reasonable figure' used to be £5 a time, but now the supply is so plentiful that a pint can be had for a sum equal to a normal week's wages i: the donor's trade."

### UNEMPLOYMENT DUE TO SICKNESS.

In these days of severe trade depression, said Mr. Greenwood, speaking at Stepney, we rarely realise the enormous wastage due to sickness. During last year the amount of sickness and disablement amongst insured persons for which benefit was paid totalled 20½ million weeks, or 394,000 years. This amount of lost time through sickness was the equivalent of the time of 394,000 fully employed workers. The unemployment due to this cause was no less than a third of the unemployment arising from bad trade. Over twice as much time was lost last year through sickness as from industrial disputes. But these appalling figures did not disclose the whole truth, as they did not include the first three days of sickness, for which no benefit was paid, nor the sickness amongst uninsured workers, and they omitted the loss of working power owing to premature deaths.

### A PALACE OF QUACKERY.

Lecturing to the Royal Society of Medicine on "Universal Cures," Professor Clark told of some extraordinary forms of quackery. He spoke of Mesmer, who had a famous tub containing an imposing collection of electrical apparatus possessing alleged marvellous powers of healing. In Paris Mesmer enjoyed the support of a large and powerful section of society, but when the Government appointed a commission to examine the apparatus it was found that no electrical effect whatever could be produced. "But Mesmer did discover something," said Professor Clark, "namely, the extraordinary effects that suggestion can produce in disease." In 1780 James Graham, who had studied medicine in Edinburgh, came to London and converted a large house in the Adelphi into one of the most remarkable palaces of quackery that have ever been known. This temple of health was furnished with the most lavish ornateness; statues, armoury, paintings, immense pillars and globes of glass, plates of burnished steel, and electrical apparatus of every description were included. In the Grand Apollo Apartment the patient sat upon a throne under a crown from which electrical discharges were produced.

### NEED OF A GOITRE SURVEY.

Walking into a chemist's shop in Switzerland, writes Dr. Saleeby in the *Daily News*, "I found bottles of 'iodised salt' upon the counter. They cost a trifle, hardly, if at all, more than we pay for table salt, in any case, and their introduction by this firm is probably due to its American associations, for iodised salt is now obtainable, if not in every State of the American Union, certainly in every State of what is called the 'goitre belt.' We need a thorough and systematic goitre survey of the children of this country; and doubtless Sir George Newman will be initiating such a survey as soon as may be feasible. Meanwhile we have tquite enough American and Swiss experience upon which to act. It matters not how our children get the iodine of which our unnatural dietary deprives them. On the whole, the most popular vehicle in Switzerland is not salt, but chocolate.

